# Virtual Stenting Based on Fractional Flow Reserve Derived from Computed Tomography in Predicting Post-Percutaneous Coronary Intervention Functional Outcomes: A Retrospective Cohort Study

**DOI:** 10.3390/jcdd12090373

**Published:** 2025-09-22

**Authors:** Han Zhao, Yanlong Ren, Jiang Li, Mingduo Zhang, Lijun Zhang, Rongliang Chen, Jia Liu, Zhengzheng Yan, Xiantao Song

**Affiliations:** 1 Department of Cardiology, Beijing Anzhen Hospital, Capital Medical University, Beijing 100029, China; zhaohanmail@163.com (H.Z.); lijiang760205@163.com (J.L.);; 2 Department of Radiology, Beijing Anzhen Hospital, Capital Medical University, Beijing 100029, China; 3 Shenzhen Institutes of Advanced Technology, Chinese Academy of Sciences, Shenzhen 518055, Chinajia.liu@siat.ac.cn (J.L.); zz.yan@siat.ac.cn (Z.Y.)

**Keywords:** fractional flow reserve derived from computed tomography, virtual stenting, fractional flow reserve, percutaneous coronary intervention

## Abstract

With the advancement of fractional flow reserve (FFR) derived from computed tomography (FFR_CT_), virtual stenting technology has gradually developed. This study investigated the performance of virtual stenting based on FFR_CT_ in predicting post-percutaneous coronary intervention (PCI) FFR. Data from 75 patients (78 blood vessels) was collected retrospectively. We randomly allocated the participants to discovery (*n* = 26) and validation (*n* = 52) cohorts. The FFR_CT_ was calculated using pre-PCI coronary computed tomography angiography images. Virtual stent implantation was simulated using blinded and non-blinded virtual stenting methods to obtain post-virtual stenting FFR_CT_. The median FFR_CT_ before PCI and invasive FFR were 0.70 (0.60–0.77) and 0.69 (0.63–0.76), respectively. The median FFR_CT_ were 0.91 (0.86–0.95) and 0.91 (0.87–0.94) in the blinded and non-blinded groups, respectively; the invasive post-PCI FFR was 0.90 (0.88–0.93). The difference between the FFR_CT_ after using the blinded/non-blinded method and the invasive post-PCI FFR were 0.010 (95% limits of agreement: −0.064 to 0.084) and 0.009 (−0.050 to 0.068) in the discovery cohort and −0.005 (−0.075 to 0.064) and −0.0002 (−0.064 to 0.064) in the validation cohort, respectively. Virtual stenting technology based on FFR_CT_ can effectively predict functional outcomes after PCI and could be a reliable tool for PCI procedural planning.

## 1. Introduction

Invasive coronary angiography (ICA) is widely regarded as the gold standard for the diagnosis of coronary heart disease. However, traditional percutaneous coronary intervention (PCI) relies on intraoperative imaging results and operator experience, which have subjectivity and limitations. Fractional flow reserve (FFR) is the gold standard for determining the presence of functional ischemia in coronary arteries. The use of FFR to guide revascularization has been verified in multiple clinical trials [1,2,3,4,5,6]. Recently, with the rapid development of medical imaging technology and computational fluid dynamics (CFD), FFR derived from computed tomography (FFR_CT_) has emerged. By analyzing coronary computed tomography angiography (CCTA) images and simulating blood flow, this method can noninvasively assess the functional significance of coronary artery stenosis [7,8,9,10,11]. Therefore, virtual coronary stenting technology further expands the application scope of FFR_CT_. This technology predicts the impact of different stent sizes and positions on postoperative hemodynamics by simulating the stent implantation process using a computer, thereby optimizing the procedural plan. In recent years, some studies have explored the application potential of virtual stenting technology in PCI procedural planning [12,13,14]. However, the above-mentioned studies employed either blinded or non-blinded virtual stenting methods for analysis. Therefore, this study aimed to assess the performance of virtual stenting technology based on the FFR_CT_ in predicting post-PCI FFR through the above two methods (blinded and non-blinded virtual stenting methods).

## 2. Materials and Methods

### 2.1. Study Population and Design

This retrospective cohort study was conducted at a single center. Between January 2018 and March 2022, consecutive patients from Beijing Anzhen Hospital were retrospectively enrolled. The inclusion criteria were patients who underwent coronary angiography, invasive FFR testing, and coronary stent placement (target vessel FFR ≤ 0.8) and those who underwent CCTA within 60 days before coronary angiography. The exclusion criteria related to specific clinical conditions were acute myocardial infarction, acute heart failure or chronic heart failure classified as New York Heart Association classes III–IV, previous stent implantation or in-stent restenosis in the target vessel, history of coronary artery bypass grafting, target vessel diameter < 2.25 mm, completely occluded lesions, and complications related to the intervention that occurred during the procedure. Exclusion criteria related to image quality or technical factors were loss of image record and poor image quality (such as severe artifacts, image discontinuity, excessive noise, incomplete coronary artery/myocardium visualization, indistinct boundaries, severe diffuse coronary calcification defined as total Agatston score ≥ 1000 or single-vessel score ≥ 500, or operator-assessed severe border obscuration) that affects accurate analysis [15,16,17]. The research protocol was approved by the Medical Ethics Committee of the Beijing Anzhen Hospital (KS2023046) and registered in the Chinese Clinical Trial Registry (ChiCTR2300078393).

### 2.2. Clinical Data-Collection Methods

Patient information was collected through the inpatient medical record system and included data on patient characteristics (such as sex, age, body mass index, and medical history), baseline examinations and laboratory results at admission, CCTA imaging, coronary angiography, invasive FFR, coronary stent implantation, and relevant medications administered. ICA, invasive FFR, and PCI were performed using standard procedures [18,19].

### 2.3. Coronary Reconstruction and Virtual Stent Deployment

CCTA was performed within 60 days before PCI (median interval of 6 days). CCTA images were obtained using a dual-source computed tomography (CT) scanner or a 256-slice CT scanner (Somatom Definition Flash, Siemens Healthcare, Forchheim, Germany; Revolution CT, GE Healthcare, Milwaukee, WI, USA). The scanner had a pixel matrix of 512 × 512, and slice thickness of 0.6 mm or 0.625 mm. The noninvasive FFR analysis and virtual stent deployment were conducted at Shenzhen Institutes of Advanced Technology. The operator was unaware of the invasive FFR measurement data. Images acquired at the end diastole (approximately 75% of the R-R interval) were used for vascular reconstruction. Patient-specific three-dimensional (3D) geometric models of the coronary arteries were then reconstructed using 3D region growing provided by Mimics software (Mimics Research 21.0, Materialise NV, Leuven, Belgium). Subsequently, two experienced cardiologists examined and manually adjusted the reconstructed geometric models if inconsistencies were identified between the model and original CCTA images, ensuring the highest possible accuracy and fidelity of reconstruction to the anatomical structure. The computational domain for CFD was discretized using an unstructured tetrahedral mesh generated by ANSYS ICEM CFD meshing software (ICEM CFD, Version 14.5. ANSYS, Inc. Canonsburg, PA, USA). The maximum element sizes were set to 0.5 mm, with each case containing over 2 million elements in total. Near the stenosis area, the mesh size was refined to 0.1 mm to accurately capture the detailed features of the blood flow dynamics [20].

The virtual stenting process involved modifying the coronary stenosis based on non-invasive FFR measurements, geometric characteristics of the lesion, and specifications of clinically available stents. The quantity, size, and location of virtual stents were carefully considered. Specifically, non-invasive FFR was first computed from CCTA-derived coronary models, which enabled precise localization of translesional pressure drops and objective quantification of lesion severity [7]. Based on both the FFR gradient and the morphological narrowing of the vessel, the target segment for virtual stent placement was identified. A single stent was implanted except for serial or diffuse long lesions. When multiple stents were connected, they were required to overlap by at least 1 mm. The center point of the virtual stent was aligned with that of the lesion to ensure adequate coverage and optimal deployment. The proximal and distal reference areas/diameters were obtained from morphologically and hemodynamically normal vessel sites, and these measurements were used as the reference for determining stent dimensions. This strategy has been validated in prior studies integrating CCTA-derived FFR with virtual stenting [12]. Furthermore, a smooth treatment was applied at the connection between the ends of the virtual stent and the vessel to simulate the elasticity factor of the vessel wall and ensure a seamless transition between the stented and non-stented segments. In this study, virtual stent implantation was simulated using both blinded and non-blinded (actual clinical stent size and position) virtual stenting methods.

### 2.4. Non-Invasive FFR Analysis

A hybrid 3D computational fluid dynamics method coupled with an integrated parameter model was utilized to compute the FFR derived from CCTA images. In this study, blood was assumed to be a viscous, laminar, and incompressible Newtonian fluid with a constant density of ρ=1.050 g/cm^3^ and constant viscosity of μ=0.035 cm^2^/s. The 3D, unsteady, and incompressible Navier–Stokes equations governing coronary blood flow can be described as

$$\rho \left(\frac{\partial u}{\partial t}+u\cdot \nabla u\right)-\nabla \cdot \sigma =f\;\;\;\;\;\;\;\;\mathrm{in}\;\;\;\;\;\;\;\;\;\Omega ,\;\nabla \cdot u=0\;\;\;\;\;\;\;\;\mathrm{in}\;\;\;\;\;\;\;\;\Omega ,$$

where u is the blood flow velocity vector, p represents the blood pressure, $\sigma =-pI+\mu (\nabla u+(\nabla u{)}^{T})$ is the Cauchy stress tensor, I is a 3×3 identity matrix, ***f*** is the external force, and Ω denotes the blood flow domain bounded by the vascular wall $\Gamma_{W}$, aortic inlet $\Gamma_{I}$, and aortic and coronary outlets $\Gamma_{O}$.

A non-slip boundary condition is enforced on $\Gamma_{W}$ assuming a rigid wall. Under allometric scaling laws, a time-varying volume flow rate $Q_{in}$ derived from the patient-specific myocardial volume, Vm, extracted from the CCTA image, was used as the inflow boundary condition. The $Q_{in}$ profile is determined by using a variant of the aortic flow waveform introduced in a previous study [21]. A transient three-element Windkessel model introduced in another study [22] is applied to the outlet boundary $\Gamma_{O}$, and the resistance and capacitance of each coronary artery branch outlet are distributed according to Murray’s law.

**Figure 1:**
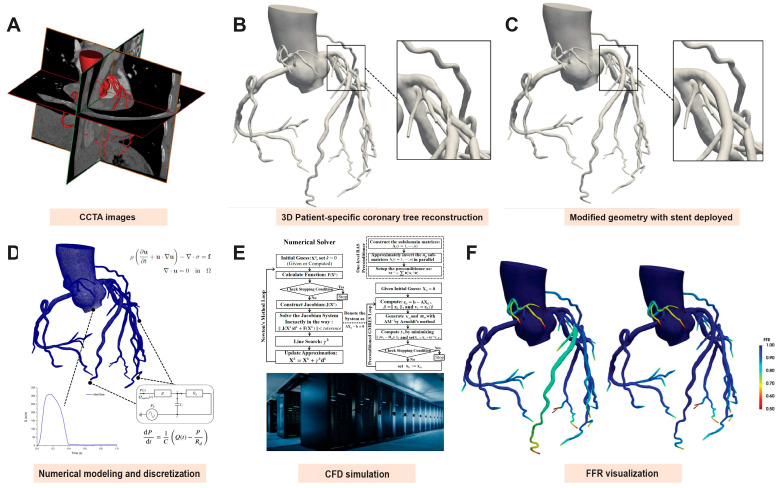
Computational workflow for virtual stent planning and hemodynamic assessment. (**A**) Coronary computed tomography angiography (CCTA) data acquisition; (**B**) Three-dimensional (3D) reconstruction of patient-specific coronary artery geometry; (**C**) Virtual stenting: replacement of stenotic regions with idealized stent geometries; (**D**) Computational setup: mathematical modeling of blood flow (Navier–Stokes equations), boundary condition assignment, and domain discretization (finite-element mesh generation); (**E**) Computational fluid dynamics (CFD) simulation: high-performance parallel computing implementation and supercomputing platform utilization; and (**F**) Post-processing to derive noninvasive fractional flow reserve (FFR) indices.

The Navier–Stokes equations were discretized using the P1-P1 stabilized finite element method in space and a fully implicit second-order backward differentiation method in time. The resulting nonlinear system was solved using the Newton–Krylov–Schwarz method [20], a highly scalable parallel solver for nonlinear partial differential equations, on a supercomputer with 240 central processing unit parallelisms. Pressure values proximal and distal to the stenosis were quantified and retrieved from the CFD model using the ParaView software (Version 5.9.1, Kitware Inc., Clifton Park, NY, USA). The detailed calculation methods for setting the boundary conditions were consistent with the computational methods published in a previous study [23]. Figure 1 shows the computational workflow for the virtual stent planning and hemodynamic assessment. The processing time for each case was approximately 20 min.

### 2.5. Statistical Analysis

Our study aimed to explore the performance of virtual stenting technology based on FFR_CT_ in predicting invasive FFR after PCI. The sample size was calculated using Power Analysis and Sample Size (PASS) software (version 15.0.5, NCSS, LLC., Kaysville, UT, USA) based on the intraclass correlation coefficient (ICC). A random sample of 19 participants who are assessed two times each produces a two-sided 80% confidence interval with a width of 0.389 when the estimated intraclass correlation is 0.600. The data was analyzed using a one-way random-effects analysis of variance model. Considering a 20% dropout rate, a minimum of 24 cases was required.

Continuous variables that follow a normal distribution are presented as mean ± standard deviation. Variables that did not follow a normal distribution are presented as median with interquartile range. Categorical variables are presented as frequency and percentage (*n* [%]). Continuous data that followed a normal distribution were compared between the groups using t-test. The Wilcoxon rank-sum test was used to assess continuous data that did not conform to normal distribution. To assess the differences in categorical variables across groups, chi-squared test or Fisher’s exact test was employed. In the analysis of diagnostic efficacy, with invasive FFR as the gold standard, a post-PCI FFR ≤ 0.9 was defined as positive (functional failure), and post-PCI FFR > 0.90 was defined as negative (functional success) [24,25,26]. The same diagnostic threshold was used for FFR_CT_. The accuracy, sensitivity, specificity, positive predictive value (PPV), and negative predictive value (NPV) of the new method (blinded/non-blinded virtual stenting method), along with their 95% confidence intervals (CIs), were obtained by comparing the new method with the gold standard through a 2 × 2 table. Agreement between the two diagnostic methods was assessed using Cohen’s kappa coefficient (κ) for categorical variables. The κ values were interpreted as follows: <0.40, poor; 0.40–0.60, moderate; 0.60–0.80, good; and >0.80, excellent. Differences in dichotomized FFR predictions between blinded and non-blinded virtual stenting methods were evaluated using McNemar’s test. Spearman’s correlation coefficient was used to evaluate the association between FFR_CT_ and invasive FFR. Bland–Altman analysis and intraclass correlation coefficient (ICC) were used to evaluate the concordance between FFR_CT_ and invasive FFR. We utilized Passing–Bablok regression analysis to assess the systematic biases of FFR_CT_ with invasive FFR and calculated the slope (proportional bias) and intercept (fixed bias) of the regression equation. We also used the root mean square error (RMSE) analysis to evaluate the magnitude of error in the FFR_CT_ prediction of the invasive FFR. During the stratified validation analysis, all participants were randomly divided into discovery (*n* = 26) and validation (*n* = 52) cohorts at a ratio of 1:2 according to the vascular level. Statistical analyses were conducted using SPSS statistical software version 25 (IBM Corp., Armonk, NY, USA) and MedCalc Statistical Software, version 22 (MedCalc Software Ltd., Ostend, Belgium). Statistical significance was set at *p* < 0.05.

## 3. Results

### 3.1. Baseline Characteristics

**Figure 2:**
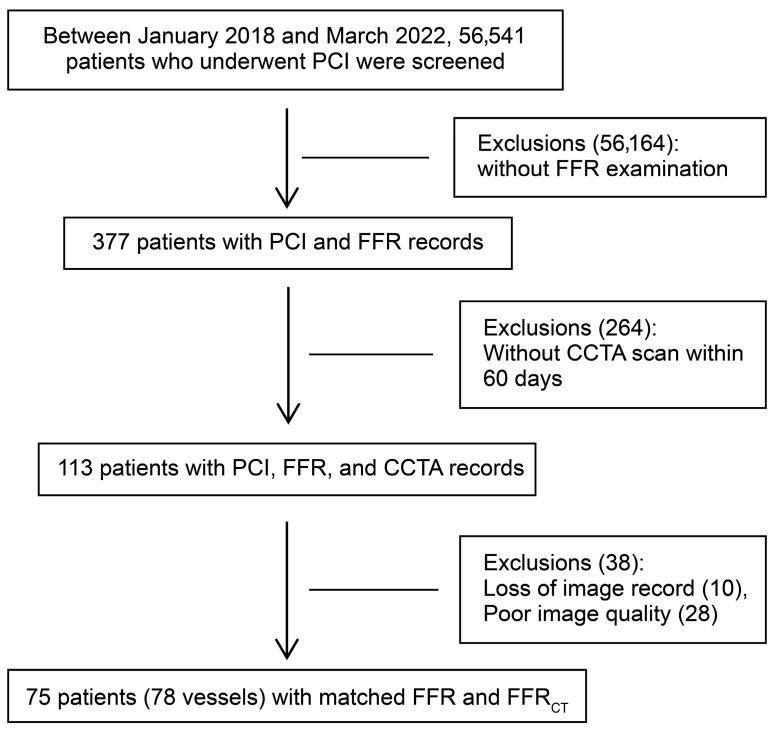
Flowchart of patient enrollment. PCI, percutaneous coronary intervention; FFR, fractional flow reserve; CCTA, coronary computed tomography angiography; FFR_CT_, fractional flow reserve derived from computed tomography.

Between January 2018 and March 2022, among the 56,541 patients screened, 56,164 patients without FFR examination records and 264 patients without CCTA images within 60 days before PCI were excluded. Further, 38 patients were excluded due to image quality issues. Although all predefined exclusion criteria were applied, no patients with any of the specific clinical conditions (acute myocardial infarction, acute heart failure or chronic heart failure classified as New York Heart Association classes III–IV, previous stent implantation or in-stent restenosis in the target vessel, history of coronary artery bypass grafting, target vessel diameter < 2.25 mm, completely occluded lesions, and complications related to the intervention that occurred during the procedure) were present in the final cohort after screening. Thus, a total of 75 patients (78 vessels, three patients contributed two vessels each) were included in the analysis. Figure 2 shows a flowchart of the patient enrollment process. Table 1 shows baseline patient data.

### 3.2. Overall Agreement Analysis

**Figure 3:**
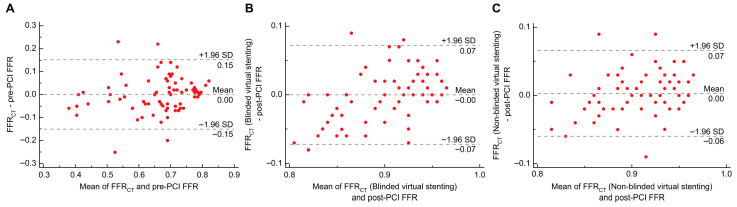
Relationship between FFR_CT_ and invasive FFR. (**A**) Relationship between FFR_CT_ and pre-PCI FFR; (**B**) Relationship between FFR_CT_ (blinded virtual stenting) and post-PCI FFR; and (**C**) Relationship between FFR_CT_ (non-blinded virtual stenting) and post-PCI FFR. PCI, percutaneous coronary intervention; FFR, fractional flow reserve; FFR_CT_, fractional flow reserve derived from computed tomography; SD, standard deviation.

In the pre-PCI assessment, the median values of preoperative FFR_CT_ and invasive FFR were 0.70 (0.60–0.77) and 0.69 (0.63–0.76), respectively, with no statistically significant difference (*p* = 0.904). Bland–Altman analysis indicated that the average discrepancy was 0.0005 (*p* = 0.953), and the 95% limits of agreement (LOA) was −0.151 to 0.152 (Figure 3A).

**Figure 4:**
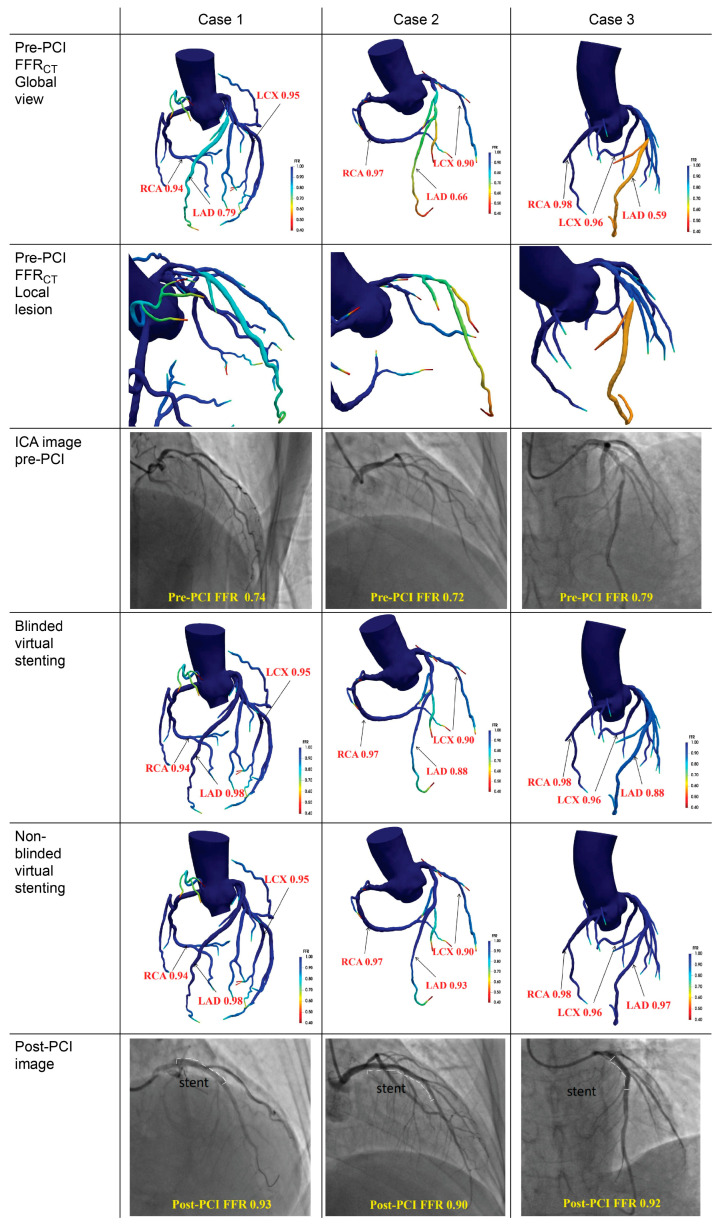
Three representative cases of virtual stent implantation. PCI, percutaneous coronary intervention; FFR, fractional flow reserve; FFR_CT_, fractional flow reserve derived from computed tomography; LAD, left anterior descending; LCX, left circumflex; RCA, right coronary artery; ICA, invasive coronary angiography.

In the post-PCI assessment, the median values of FFR_CT_ in the blinded virtual stenting group did not exhibit a statistically significant difference when compared to the post-PCI FFR values (0.91 [0.86–0.95] vs. 0.90 [0.88–0.93], *p* = 0.908). Similarly, no statistically significant difference was observed in the median values of FFR_CT_ in a non-blinded virtual stenting group compared with the post-PCI FFR (0.91 [0.87–0.94] vs. 0.90 [0.88–0.93], *p* = 0.347). Bland–Altman analysis revealed that the average discrepancy between FFR_CT_ after blinded virtual stenting and post-PCI FFR was −0.0001 (*p* = 0.976, 95% LOA: −0.072 to 0.072; Figure 3B). For the non-blinded virtual stenting group, the mean difference between FFR_CT_ and post-PCI FFR was 0.003 (*p* = 0.421,95% LOA: −0.060 to 0.066; Figure 3C). Figure 4 shows the three representative cases of virtual stent implantation.

### 3.3. Diagnostic Efficacy and Cohort-Stratified Validation

#### 3.3.1. Diagnostic Efficacy

The analysis of diagnostic efficacy was at the vascular level (*n* = 78). The accuracy of the blinded virtual stenting method in predicting functional failure after PCI was 79.5% (95% CI: 68.8–87.8%), with sensitivity 75.6% (95% CI: 59.7–87.6%), specificity 83.8% (95% CI: 68.0–93.8%), PPV 83.8% (95% CI: 70.9–91.6%), and NPV 75.6% (95% CI: 64.0–84.4%). The κ value was 0.59 (95% CI: 0.41–0.77, *p* < 0.001; Table S1). The accuracy of the non-blinded virtual stenting method in predicting functional failure after PCI was 78.2% (95% CI: 67.4–86.8%), with sensitivity 73.2% (95% CI: 57.1–85.8%), specificity 83.8% (95% CI: 68.0–93.8%), PPV 83.3% (95% CI: 70.1–91.4%), and NPV 73.8% (95% CI: 62.5–82.7%). The κ value was 0.57 (95% CI: 0.38–0.75, *p* < 0.001; Table S2). Both methods had high specificity, PPV, and acceptable sensitivity. The agreements between the two methods and gold standard were moderate. The blinded and non-blinded virtual stenting methods showed good agreement in dichotomized post-PCI FFR classifications among all 78 vessels (McNemar’s test, *p* = 1.0), with symmetric discordances (6 vs. 7) in 16.7% of cases.

#### 3.3.2. Cohort-Stratified Validation

**Figure 5:**
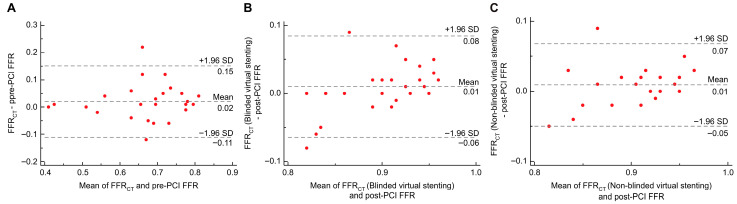
Relationship between FFR_CT_ and invasive FFR of the discovery cohort. (**A**) Relationship between FFR_CT_ and pre-PCI FFR; (**B**) Relationship between FFR_CT_ (blinded virtual stenting) and post-PCI FFR; and (**C**) Relationship between FFR_CT_ (non-blinded virtual stenting) and post-PCI FFR. PCI, percutaneous coronary intervention; FFR, fractional flow reserve; FFR_CT_, fractional flow reserve derived from computed tomography; SD, standard deviation.

In the discovery cohort (*n* = 26), the correlation coefficient between FFR_CT_ and invasive pre-PCI FFR was 0.825 (*p* < 0.001); the correlation coefficients between blinded and non-blinded virtual stenting FFR_CT_ and invasive post-PCI FFR were 0.767 (*p* < 0.001) and 0.859 (*p* < 0.001), respectively. The ICC of FFR_CT_ and invasive pre-PCI FFR was 0.824 (*p* < 0.001), with a 95% confidence interval (CI) of 0.646–0.917. The ICCs for FFR_CT_ and invasive post-PCI FFR after blinded and non-blinded virtual stenting were 0.691 (*p* < 0.001, 95% CI: 0.421–0.849) and 0.768 (*p* < 0.001, 95% CI: 0.547–0.889), respectively. The Bland–Altman analysis showed that the mean difference between the FFR_CT_ and pre-PCI FFR was 0.020 (*p* = 0.140, 95% LOA: −0.111 to 0.151; Figure 5A). The differences between FFR_CT_ after using the blinded and non-blinded methods of virtual stenting and the invasive post-PCI FFR were 0.010 (*p* = 0.191, 95% LOA: −0.064 to 0.084) and 0.009 (*p* = 0.130, 95% LOA: −0.050 to 0.068), respectively (Figure 5B,C).

**Figure 6:**
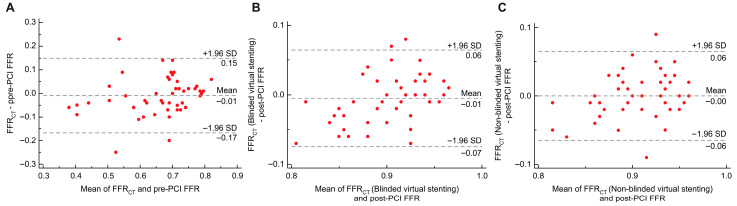
Relationship between FFR_CT_ and invasive FFR of the validation cohort. (**A**) Relationship between FFR_CT_ and pre-PCI FFR; (**B**) Relationship between FFR_CT_ (blinded virtual stenting) and post-PCI FFR; and (**C**) Relationship between FFR_CT_ (non-blinded virtual stenting) and post-PCI FFR. PCI, percutaneous coronary intervention; FFR, fractional flow reserve; FFR_CT_, fractional flow reserve derived from computed tomography; SD, standard deviation.

In the validation cohort (*n* = 52), the correlation coefficient between FFR_CT_ and invasive pre-PCI FFR was 0.702 (*p* < 0.001), and the correlation coefficients between blinded and non-blinded virtual stenting FFR_CT_ and invasive post-PCI FFR were 0.692 (*p* < 0.001) and 0.688 (*p* < 0.001), respectively. The ICC of FFR_CT_ and pre-PCI invasive FFR was 0.759 (*p* < 0.001), with a 95% CI of 0.615–0.854. The ICCs for the FFR_CT_ and invasive post-PCI FFR after blinded and non-blinded virtual stenting were 0.660 (*p* < 0.001, 95% CI: 0.474–0.789) and 0.662 (*p* < 0.001, 95% CI: 0.477–0.791), respectively. In the Bland–Altman analysis, the mean difference between the FFR_CT_ and pre-PCI FFR was −0.009 (*p* = 0.413, 95% LOA: −0.167 to 0.149; Figure 6A). The difference in FFR_CT_ after blinded virtual stenting and invasive post-PCI FFR was −0.005 (*p* = 0.297, 95% LOA= −0.075 to 0.064), and the difference between FFR_CT_ after non-blinded virtual stenting and invasive post-PCI FFR was −0.0002 (*p* = 0.967, 95% LOA= −0.064 to 0.064; Figure 6B,C).

### 3.4. Regression Models and Error Analysis

#### 3.4.1. Passing–Bablok Regression

**Figure 7:**
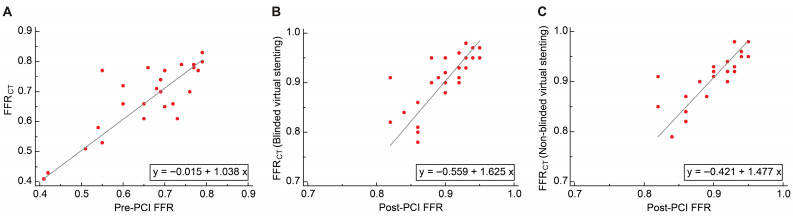
Passing–Bablok regression of the discovery cohort. (**A**) FFR_CT_ and pre-PCI FFR; (**B**) FFR_CT_ (blinded virtual stenting) and post-PCI FFR; and (**C**) FFR_CT_ (non-blinded virtual stenting) and post-PCI FFR. PCI, percutaneous coronary intervention; FFR, fractional flow reserve; FFR_CT_, fractional flow reserve derived from computed tomography.

In the discovery cohort, FFR_CT_ showed good consistency with the pre-PCI FFR; the slope was 1.038 (95% CI: 0.828–1.357), and the intercept was −0.015 (95% CI: −0.219–0.138; Figure 7A). A positive proportional bias was observed between FFR_CT_ after blinded and non-blinded virtual stenting and post-PCI FFR; slopes were 1.625 (95% CI: 1.000–2.111) and 1.477 (95% CI: 1.000–1.727), respectively. The fixed bias between FFR_CT_ after blinded and non-blinded virtual stenting and post-PCI FFR were not significant; the intercepts were −0.559 (95% CI: −1.003 to 0.010) and −0.421 (95% CI: −0.649 to 0.015), respectively (Figure 7B,C).

**Figure 8:**
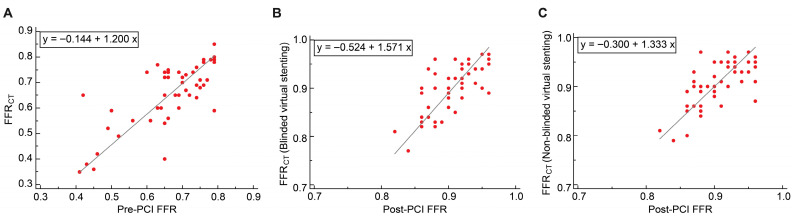
Passing–Bablok regression of the validation cohort. (**A**) FFR_CT_ and pre-PCI FFR; (**B**) FFR_CT_ (blinded virtual stenting) and post-PCI FFR; and (**C**) FFR_CT_ (non-blinded virtual stenting) and post-PCI FFR. PCI, percutaneous coronary intervention; FFR, fractional flow reserve; FFR_CT_, fractional flow reserve derived from computed tomography.

In the validation cohort, FFR_CT_ showed positive proportional bias with the pre-PCI, the slope was 1.200 (95% CI: 1.000–1.462), the fixed bias was not significant, and the intercept was −0.144 (95% CI: −0.325 to 0.005; Figure 8A). A positive proportional bias was observed between FFR_CT_ following both blinded and non-blinded virtual stenting and post-PCI FFR; slopes were 1.571 (95% CI: 1.222–2.000) and 1.333 (95% CI: 1.000–1.667), respectively. A negative fixed bias was observed between FFR_CT_ after blinded and non-blinded virtual stenting and post-PCI FFR; the intercepts were −0.524 (95% CI: −0.910 to −0.208) and −0.300 (95% CI: −0.607 to 0.000), respectively (Figure 8B,C).

#### 3.4.2. RMSE Analysis

The RMSE of the FFR_CT_ in the discovery and validation cohorts compared with the invasive pre-PCI FFR were 0.0684 and 0.0804, respectively. In the discovery cohort, the RMSEs of the FFR_CT_ after blinded and non-blinded virtual stenting and invasive post-PCI FFR were 0.0385 and 0.0309, respectively. In the validation cohort, the RMSEs of the two methods were 0.0355 and 0.0327, respectively.

## 4. Discussion

Our findings revealed that FFR_CT_ and virtual stenting technology have good consistency with invasive FFR in preoperative and postoperative evaluations. The difference between the preoperative FFR_CT_ and invasive FFR in this study was small. Bland–Altman analysis demonstrated good overall consistency between the two measures, indicating that the FFR_CT_ accurately reflects the true coronary hemodynamic status at the overall level. Further, the differences between FFR_CT_ and invasive FFR in both the discovery and validation cohorts were minimal; the correlation coefficients and ICCs showed good inter-group consistency, thereby confirming the reliability of FFR_CT_ in preoperative functional assessment. This result is consistent with those of the DISCOVER-FLOW study [7] (r = 0.717 for the correlation between FFR_CT_ and invasive FFR) and the NXT trial [9] (r = 0.82). The slightly wider LOA in the Bland–Altman analysis and the slightly larger RMSE suggest significant differences between FFR_CT_ and invasive FFR at the individual level, which may be related to factors such as computed tomography (CT) image calcification artifacts. For individual cases, especially when the FFR_CT_ value is close to 0.75–0.80, further evaluation by combining intravascular imaging (such as optical coherence tomography [OCT] and intravascular ultrasound [IVUS]) and clinical conditions is recommended.

The postoperative assessment revealed no significant differences in FFR_CT_ between the blinded and non-blinded virtual stenting methods and invasive FFR. Bland–Altman analysis also demonstrated good consistency between blinded and non-blinded virtual stenting methods and invasive post-PCI FFR, with mean differences close to zero and narrow limits of agreement, indicating that the virtual stenting algorithm can effectively simulate hemodynamic changes after stent implantation, consistent with the findings of earlier studies by Kim et al. [12] (mean difference after intervention: 0.024, 95%; LOA: −0.08 to 0.13). The LOA range of the non-blinded method was narrower, suggesting that the non-blinded virtual stenting technique predicted smaller individual differences with higher consistency. The input of actual stent parameters could further reduce the prediction error of the model, reflecting the optimization and adjustment of the stent size and position based on the operator’s experience.

The diagnostic efficacy results suggest that when FFR >0.9 after PCI is used as the functional success criterion, both the blinded and non-blinded virtual stenting techniques have relatively high specificity in predicting the post-PCI outcome. The accuracy of both approaches was close to 80%, indicating that the software algorithm for autonomously selecting stent parameters may have the potential to be independent of operator experience. However, their sensitivity is relatively low, suggesting that approximately 25% of cases with functional failure (FFR ≤ 0.90) may be missed. Combined with the Passing–Bablok regression results (the slopes for both blinded and non-blinded methods were slightly greater than 1), this indicates that FFR_CT_ may tend to overestimate high FFR values (>0.9) on prediction, and conducting a comprehensive assessment by combining intravascular imaging (such as OCT and IVUS) and clinical indicators is advisable. The PPVs of both the blinded and non-blinded methods exceeded 83%, which is higher than the 50% reported by Kim et al. [12] in their earlier study (with a cut-off value of FFR ≤ 0.8). This discrepancy may be attributed to the higher FFR threshold (0.9) selected in this study, which is in line with the recent consensus that postoperative FFR ≤ 0.9 indicates a poor prognosis [24,25,26].

In the stratified validation of the discovery cohort, the correlation coefficients and ICCs of FFR_CT_ between blinded and non-blinded virtual stenting and invasive post-PCI FFR indicated moderate to good inter-group consistency. In the validation cohort, the correlation coefficients decreased slightly, which may be attributed to lesion heterogeneity and physiological differences between virtual simulation and post-PCI hemodynamics. Nevertheless, the ICCs remained at a moderate level, and the stability in the validation cohort supported the reliability of the model. In the Bland–Altman analysis, the average differences between the blinded and non-blinded virtual stenting postoperative FFR_CT_ and the invasive post-PCI FFR were both small in the discovery and validation cohorts. This indicates that, in both cohorts, the virtual stenting postoperative FFR_CT_ using these two methods is consistent with the invasive post-PCI FFR. This result supports the generalizability of virtual stenting technology in different populations. In the Passing–Bablok regression, the preoperative regression slope of the discovery cohort was close to 1, and the intercept was close to 0, suggesting that preoperative FFR_CT_ and invasive FFR had good consistency, without significant systematic bias. The regression slope of the validation cohort was slightly higher. This indicates that the complexity differences in lesions or sample size differences between cohorts have an impact on model stability. Preoperative FFR_CT_ may overestimate cases with higher FFR values (close to 0.80). Postoperative analysis revealed that the regression slopes of the blinded virtual stenting method in both the discovery and validation cohorts were >1, suggesting that the FFR_CT_ may overestimate the true value when the invasive FFR is relatively high. The intercepts in the validation cohort were <0, indicating that the model may generally underestimate the invasive FFR. The regression slope of the non-blinded virtual stenting method was lower than that of the blinded method, and the intercept was closer to 0. The CIs of the slopes and intercepts were narrower. Moreover, the RMSE of the non-blinded group was lower than that of the blinded group in both cohorts, indicating that the input of the stent parameters (such as diameter and length) partially corrected the model and improved the accuracy of the simulation. Although the model shows a certain degree of correlation with FFR, the systematic bias emphasizes that even a well-correlated computational model may need calibration when applied to a new cohort. This highlights the importance of further validation before clinical application. A certain degree of simplification of the hemodynamic effects is possible, which can be further optimized by incorporating additional influencing factors (such as plaque composition, local shear stress changes, and microcirculation resistance). Clinically, intravascular imaging (such as OCT/IVUS) and clinical indicators can be combined for a comprehensive assessment [13].

Recently, studies have been conducted on the calculation and simulation of coronary stent implantation based on the quantitative flow ratio (QFR) [27] and virtual FFR (vFFR) [28]. All these studies found that the procedural planning results using QFR and vFFR have good correlation and consistency with post-PCI FFR. However, both these procedural planning methods need to be performed during ICA; thus, they are not suitable for planning before invasive procedures. In comparison, modeling based on CCTA images and virtual stent implantation seem to have more advantages. Future studies should directly compare FFR_CT_-based virtual stenting with other computational methods (such as QFR/vFFR) using standardized endpoints and workflow metrics to further establish its relative clinical utility.

PCI in patients with atrial fibrillation (AF) presents unique challenges in clinical practice. These patients often face a dual threat of procedure-related ischemic risks and medication-induced bleeding risks, necessitating comprehensive strategies to optimize therapeutic outcomes. Strict adherence to appropriate indications for invasive diagnostics and revascularization procedures is crucial for improving prognosis [29]. In this context, preprocedural CCTA plays a pivotal role; by accurately assessing coronary anatomy and lesion characteristics, combined with functional evaluations such as FFR_CT_ and simulation of post-PCI outcomes, it can effectively avoid unnecessary invasive procedures and PCI, thereby reducing patient exposure to these dual risks. This approach provides new insights for improving clinical outcomes of PCI in patients with AF.

### 4.1. Innovativeness and Strengths

This study confirmed the good generalization performance of the virtual stenting model through a stratified analysis of the discovery and validation cohorts. The predictive performances of blinded and non-blinded virtual stenting techniques were analyzed separately. Blinded virtual stenting, which automatically selects the stent size and position to help eliminate the subjective bias of the operator, may simplify the preoperative procedure planning process, especially for inexperienced operators or standardized lesions. The non-blinded virtual stenting strategy may be closer to clinical needs, simulating the effects of different stenting strategies preoperatively and optimizing the procedure plan, thus becoming a reliable tool for individualized PCI planning.

### 4.2. Limitations

All cases in the present investigation were from a single center, which may have led to significant selection bias. These results need to be verified through multicenter studies to ensure generalizability. Because this was a retrospective study, many cases were excluded because of incomplete data or poor CCTA image quality, resulting in a small sample size, both for discovery and validation cohorts, and increased selection bias. Further expansion of the study population and multi-center prospective studies are needed to enhance statistical power. The analysis of diagnostic efficacy was at the vascular level (78 vessels from 75 patients, with three patients contributing two vessels each), and a mixed-effects model was not used to account for the intra-patient clustering. Thus, the precision might be overestimated due to unadjusted clustering in 4% (3/75) of patients. Given the anatomical independence of coronary vessel involvement and the small proportion of clustered data, this approach possibly had a relatively minor impact on the overall findings. Nevertheless, future studies with larger samples should adopt hierarchical modeling to enhance statistical rigor. Although our research employed validation methods, these comprised internal validation rather than external validation. The validation cohort, though randomly split, was not powered to detect small effects. Larger-scale external validation cohort studies need to be conducted in the future to confirm these findings. While the method demonstrated 73% (75/103) feasibility in our cohort, its clinical applicability may be limited in subpopulations with poor image quality or complex coronary lesions. Future studies should focus on expanding compatibility to broader patient demographics. The current study, as an initial technical validation, was not powered for subgroup analyses. Although the technology demonstrated generally consistent results overall, significant data dispersion existed at the individual level. Further investigations involving pre-specified subgroup analyses (such as stratified by vascular lesion characteristics) and model refinement are needed to identify potential beneficiary populations. Our study did not include patients with AF; therefore, the generalizability of our findings to populations with AF requires further validation. This study lacked follow-up endpoint events (such as major adverse cardiovascular events and target vessel failure) to verify the clinical relevance of the simulation results. Further follow-up studies are warranted. The clinical implications of these findings require validation in prospective studies with hard endpoints.

## 5. Conclusions

This study verified that virtual stenting technology based on the FFR_CT_ can effectively predict functional outcomes after PCI and has the potential to become a reliable tool for PCI procedural planning.

## Figures and Tables

**Table 1:** Baseline characteristics.

Number of cases	75
Number of vessels	78
Age, years	59.76 ± 8.77
Male	56 (74.7%)
BMI	25.79 ± 3.25
Hypertension	44 (58.7%)
Diabetes mellitus	16 (21.3%)
Hyperlipidemia	38 (50.7%)
Current tobacco use	33 (44%)
Prior PCI (Non-target vessel)	1 (1.3%)
LVEF (%)	65 (61–67)
Systolic blood pressure (mmHg)	131.80 ± 15.60
Diastolic blood pressure (mmHg)	76.44 ± 9.42
Heart rate (beats per minute)	68 (64–76)
Creatinine (µmol/L)	69.60 ± 12.02
LDL-C (mmol/L)	2.15 (1.73–2.88)
Time interval between CCTA and PCI (days)	6 (2–11)
Target vessel	
LAD-PCI	58 (74.4%)
LCX-PCI	7 (9%)
RCA-PCI	13 (16.7%)
Actual stent length (mm)	26.5 (18–37.25)
Virtual stent length (mm)	23 (17–30)

BMI, body mass index; LVEF, left ventricular ejection fraction; LDL-C, low-density lipoprotein cholesterol; CCTA, coronary computed tomography angiography; PCI, percutaneous coronary intervention; LAD, left anterior descending; LCX, left circumflex; RCA, right coronary artery.

## Data Availability

The datasets generated during this study are not publicly available due to ethical restrictions protecting participant privacy.

## Supplementary Materials

The following supporting information can be downloaded at: https://www.mdpi.com/article/10.3390/jcdd12090373/s1, Table S1: Diagnostic efficacy of blinded virtual stenting method (vascular level, *n* = 78); Table S2: Diagnostic efficacy of non-blinded virtual stenting method (vascular level, *n* = 78).

## Abbreviations

The following abbreviations are used in this manuscript:

ICA	Invasive coronary angiography
PCI	Percutaneous coronary intervention
FFR	Fractional flow reserve
CFD	Computational fluid dynamics
CCTA	Coronary computed tomography angiography
FFR_CT_	Fractional flow reserve derived from computed tomography
ICC	Intraclass correlation coefficient
RMSE	Root mean square error
PPV	Positive predictive value
NPV	Negative predictive value
QFR	Quantitative flow ratio
vFFR	Virtual fractional flow reserve
OCT	Optical coherence tomography
IVUS	Intravascular ultrasound
