# Supplementary material for: Virtual Stenting Based on Fractional Flow Reserve Derived from Computed Tomography in Predicting Post-Percutaneous Coronary Intervention Functional Outcomes: A Retrospective Cohort Study
Source: J Cardiovasc Dev Dis. 2025 Sep 22;12(9):373. doi: 10.3390/jcdd12090373 (PMC12470931; doi:10.3390/jcdd12090373)
Supplement: Supplementary file 1 [file jcdd-12-00373-s001.zip › jcdd-3723221-supplementary.pdf]

Supplementary materials

Table S1. Diagnostic efficacy of blinded virtual stenting method (vascular level, n=78)

FFR_{CT} (blinded virtual stenting)	Invasive FFR (gold standard)		Total
	+(≤0.90)	-(>0.90)	
+(≤0.90)	31	6	37
-(>0.90)	10	31	41
Total	41	37	78

FFR, fractional flow reserve; FFR_{CT}, fractional flow reserve derived from computed tomography

Table S2. Diagnostic efficacy of non-blinded virtual stenting method (vascular level, n=78)

FFR_{CT} (non-blinded virtual stenting)	Invasive FFR (gold standard)		Total
	+(≤0.90)	- (>0.90)	
+(≤0.90)	30	6	36
- (>0.90)	11	31	42
Total	41	37	78

FFR, fractional flow reserve; FFR_{CT}, fractional flow reserve derived from computed tomography
